# MicroRNA profiling in dogs undergoing induced ischemic heart infarction: An experimental study

**DOI:** 10.14202/vetworld.2023.1319-1324

**Published:** 2023-06-13

**Authors:** Liqaa A. Raffee, Khaled Z. Alawneh, Musa Ahmed Mohammed Alshehabat, Hazem Haddad, Saied A. Jaradat

**Affiliations:** 1 Department of Accident and Emergency Medicine, Faculty of Medicine, Jordan University of Science and Technology, Irbid, Jordan; 2 Department of Diagnostic Radiology and Nuclear Medicine, Faculty of Medicine, Jordan University of Science and Technology, Irbid, Jordan; 3 Department of Clinical Veterinary Medical Sciences, Faculty of Veterinary Medicine, Jordan University of Science and Technology, Irbid, Jordan; 4 Princess Haya Biotechnology Center, Jordan University of Science and Technology, Irbid 22110, Jordan

**Keywords:** dog model, heart infarction, microRNAs, veterinary

## Abstract

**Background and Aim:**

MicroRNAs (miRNAs) play an important role in various biological functions. According to many studies, miRNA expression is tissue-specific, strongly controlled throughout embryogenesis, and over- or under-expressed in numerous disorders, including cardiovascular pathologies. This study aimed to screen, characterize, and profile many induced biomarkers (miRNAs) in dog serum before and after experimentally inducing a regional myocardial infarction (MI) by occluding the coronary arteries under general anesthesia.

**Materials and Methods:**

A preclinical experimental animal study recruited 12 healthy canine dogs. The selected canine dogs were anesthetized with 1 mg/kg xylazine and 15 mg/kg ketamine before undergoing femoral arterial catheterization under fluoroscopic supervision. Commercial assay kits were used to purify total RNA and miRNA before the occlusion and 2 h after the occlusion according to the manufacturer’s guidelines, and the samples were stored in RNase/DNase-free water at −80°C. Data were analyzed by GraphPad Prism 5.0 software (GraphPad Prism, San Diego, CA) SPSS, and GenEx software (www.multid.se) or (REST V3).

**Results:**

Among 325 transcribed genes, 20 were identified in 2 h. After MI, 14 biomarkers were negative, indicating downregulation, and 6 (3-F08, 3-B10, 4-A11, 1-A06, 2-E01, 3-F10) were positive, indicating upregulation. Polymerase chain reaction assay results showed a normalized fold-change in gene expression in the test sample. Fold values >1 represented a biologically significant change.

**Conclusion:**

Profiling of miRNAs before and after MI in a dog model revealed upregulation of six previously unidentified biomarkers (3-F08, 3-B10, 4-A11, 1-A06, 2-E01, and 3-F10), indicating various miRNA regulatory patterns.

## Introduction

MicroRNAs (miRNAs) are small RNA molecule sequences produced by the body. Most species may have miRNA-encoding genes, including those for viruses, in their genomes. Post-transcriptional suppression of gene expression is their main mode of action [1]. The short length (22 nt) is thought to increase target-gene specificity while minimizing non-specific effects. Approximately 30% of the genes in the human genome, which may include approximately 2000 miRNAs, are thought to be controlled by miRNAs [2]. Cardiovascular diseases (CVDs) are the leading cause of death globally, with a growing incidence of mortality and morbidity in the elderly population and are caused by genetic and epigenetic interactions [3–5]. Genomic instability, cellular senescence, telomere lengthening, signaling network, dietary restriction, molecular damage (particularly in oxidative injury), and overactivity of processes that can lead to hypertrophy-associated pathologies (hyperfunction) during adulthood, loss of proteostasis, mitochondrial dysfunction, stem-cell exhaustion, and alterations in intercellular communication are among the genetic components [6–10]. DNA methylation, post-translational histone modifications, and a family of small non-coding RNAs known as miRNAs are the most significant epigenetic alterations in mammalian cells (miRNAs or miRs) [8–11].

MicroRNAs play an important role in various biological functions. According to many studies, miRNA expression is tissue-specific, strongly controlled throughout embryogenesis, and over- or under-expressed in numerous disorders, including cardiovascular pathologies [12, 13]. Numerous miRNAs are up- or down-regulated in response to a lack of oxygen. Many dysregulated miRNAs are based on the hypoxia-inducible factor, a transcription factor crucial in the response to low oxygen. Other research has revealed that oxidative stress activates additional transcription factors that regulate several homeostatic and physiological processes, such as metabolism, angiogenesis, cell survival, oxygen delivery, and miRNA expression [14, 15]. Apoptosis, fibrosis, and multiple cell types, such as cardiomyocytes, immunological cells, fibroblasts, and endothelial cells, are all triggered in response to myocardial infarction (MI) [16]. A rat model was used in a time-course investigation of serum miR-1, including time points at 1, 3, 6, 12, and 24 h and at 3, 7, 14, 21, and 28 days after acute MI (AMI). A previous study found that serum miR-1 levels increased after AMI, peaked at 6 h, and then returned to baseline 3 days later, with a strong positive connection with MI size. MiR-1 was dramatically increased within 24 h after AMI in 31 patients with AMI and exhibited a positive connection with blood creatine kinase-MB, suggesting that its link to MI size also occurs in humans. Serum levels reverted to baseline on days 3 and 7 [17]. MicroRNA characterization and profiling is a potential technique for detecting biomarkers accompanying different diseases. Fluoroscopic assessment of urine and blood samples has demonstrated a significant role of MiRNA genetic encoding in the diagnostic, prognostic, and therapeutic evaluation of cerebral artery occlusion in dogs [18].

The role of upregulation or downregulation of novel biomarkers in coronary artery occlusion and disease consequences requires further testing in preclinical models to follow translational research. This study aimed to characterize the miRNA profiles associated with CVDs in the serum of dogs after inducing ischemia by selectively blocking coronary arteries under anesthesia. We investigated the downregulation and upregulation of biomarkers to estimate the degree of gene expression in the heart.

## Materials and Methods

### Ethical approval

The study was approved by the Animal Care and Use Committee of Jordan University of Science and Technology (Approval no. 155/2017).

### Study period and location

The study was conducted from August 2018 to August 2019 at the campus of the Jordan University of Science and Technology.

### Study design

This study was an experimental animal study conducted in a preclinical setting.

### Sample size

Twelve healthy canine dogs aged between 1 and 5 years were included in this study. Of the 12 dogs, six were the control group, and the other six were the intervention group. The animals were cared for in compliance with the Principles of Laboratory Animal Care, as described by the National Institutes of Health (National Institutes of Health publication no. 96–23, revised in 1996).

### Inclusion and exclusion criteria

Only dogs that were physically fit were included in this study. Physical fitness was determined by measuring platelets, white cells, red cells, and levels in a serum biochemistry panel, including creatinine, albumin, liver enzymes (alanine transaminase, alkaline phosphatase, and gamma-glutamyl tansferase), glucose, globulin, and urea. Dogs with a history of any health disorder were excluded, including those who were obese and aggressive.

### Experimental animals

The animals selected for the study were vaccinated for adenovirus 1, canine distemper, adenovirus 2, leptospira, rabies, and parvovirus (Vanguard^®^, New Jersey, USA). In addition, a deworming regimen using Ascaten^®^ (1 Tab./5 kg, Hong Kong) was started and repeated after 2 weeks. The dogs were housed in the hosting facility for 14 days to begin the experiment. Each dog had its collar ID and was separately housed in cages in a daily food and water facility. The dogs were fed commercially available food (Benson™, Amman, Jordan). These special treatments were given to the dogs before the experiment to help them adjust to this new environment and to absorb any new physical or mental abnormality that could not be found previously. On the day of the experiment, the canine dogs were anesthetized with morphine and α-chloralose and ventilated to hold physiological arterial blood gases. The temperature was maintained at 37°C at the time of ventilation. For the surgical procedure, sterile conditions were maintained. A median sternotomy was conducted by hooking bipolar stainless steel and Teflon-coated electrodes into the right and left atria for stimulation. The proximal left anterior descending (LAD) coronary artery was ligated below the first diagonal branch to induce MI in dogs. The chest was then opened under general anesthesia through the left fourth intercostal space, the pericardium was opened to expose the heart, and the proximal LAD coronary artery was ligated below the first diagonal branch to induce MI. An electrocardiograph was used to monitor the induction of MI and infarction size after ligation.

### Experimental procedure

Selected canine dogs were anesthetized with 1 mg/kg xylazine and 15 mg/kg ketamine before undergoing femoral arterial catheterization under fluoroscopic supervision. A strict approach was used to traverse around the coronary artery and selectively occlude it.

### Measuring miRNAs

Blood samples were collected from the cephalic vein or jugular for miRNA profiling through venipuncture. A 4-mL blood sample was collected in a 5-mL syringe (with a 22-gauge needle). A 1-mL aliquot of blood in a syringe was kept under vacuum for mild aspiration and to minimize vessel occlusion. The blood sample was collected in three (3.8%) tubes of sodium citrate at a citrate-to-blood ratio of 1:9. Carefully, the tubes were inverted repeatedly for some time, and the sample was stored at 37°C in a heating chamber until the remaining procedure was performed. From the collected samples, assay kits were used for the purification of total RNA and miRNA before the occlusion and 2 h after the occlusion according to the manufacturer’s guidelines. The samples were stored in RNase/DNase-free water at −80°C. Nano-DropTM 1000 spectrophotometer (Thermo Scientific; Massachusetts, USA) and bio-analyzer 2100 (Agilent; California, USA) were used to check the miRNA and total RNA quantity and quality. The TaqMan Human miRNA Assay Set (Applied Biosystems, Massachusetts, USA) consisting of Sanger database unique assays to quantify MI miRNAs, and ten controls (Z30 and nine different SNORs/RNUs) were used for screening a set of 20 normal specimens and 20 MIs for differentially expressed miRNAs. The TaqMan miRNA reverse transcription assay depends on using a hairpin structure with a specific primer during cDNA synthesis. Mature miRNA-specific detection probes precluded the detection of precursor miRNAs (Applied Biosystems). Specifically, various RT primers were pooled according to the suggestions of ABI.

### Statistical analysis

For each listed variable, descriptive statistics were collected. Data were analyzed by GraphPad Prism 5.0 software (GraphPad Prism, San Diego, CA) SPSS, and GenEx software (MultiD Analyses AB; www.multid.se, Göteborg Sweden) or (REST V3). A paired sample *t*-test was performed for the associated observations, with p-value of 0.05 used to determine statistical significance. The comparative cycle threshold technique was used to obtain relative quantification of miRNA expression. geNorm techniques were used to normalize miRNA expression data to relevant reference genes for accurate comparability. A serial examination of miRNAs in the blood was performed: one analysis after the occlusion (1–2 h later) and another analysis before the arterial occlusion.

### Location and safety considerations

The dogs were housed at the Jordan University of Science and Technology Veterinary Health Center to meet the study requirements. Each dog was kept in a cage with food and water requirements. To eliminate any zoonotic risks, the animals were thoroughly vaccinated. Portable fluoroscopy devices were used in the housing at the veterinary teaching hospital during the investigation.

## Results

**Figure-1:**
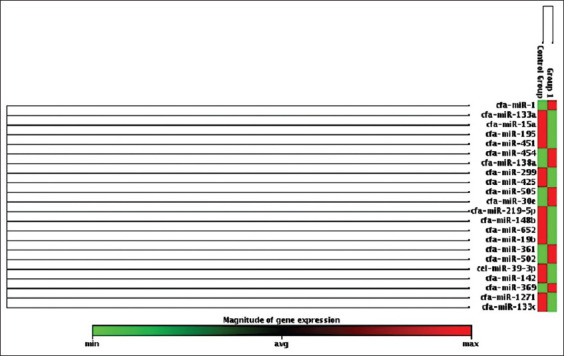
Box chart showing significant fold-change between the control group and group 1 in heart.

**Figure-2:**
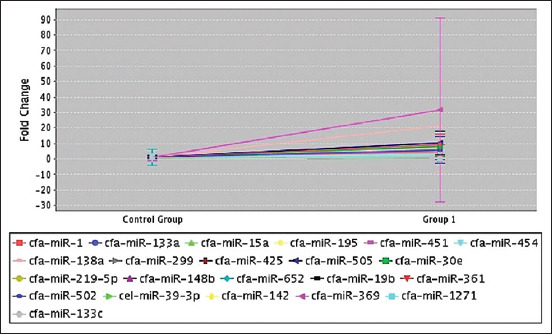
Bar graph showing significant fold-change between control group and group 1 in heart.

**Figure-3:**
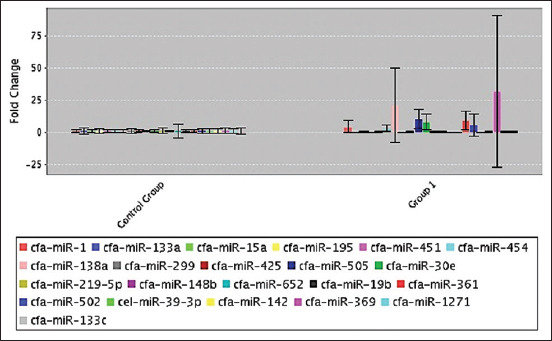
Magnitude of gene expression in heart.

Serum samples from 12 dogs were assayed by polymerase chain reaction to measure fold-change and fold-regulation values. A list of genes and plate positions for heart miRNAs in serum is presented in Table-1. The findings showed the least calculated and reported fold-change value because the average gene threshold cycle was comparatively higher. The fold-change results showed greater variations but were not statistically significant. Upregulation was indicated by an increased fold-change value (a positive change). On the contrary, downregulation was indicated by a decreased fold-change value (a negative change) of <1. Among 325 transcribed genes, 20 were identified in 2 h. After MI, 14 biomarkers were negative, indicating downregulation, and 6 (3-F08, 3-B10, 4-A11, 1-A06, 2-E01, and 3-F10) were positive, indicating upregulation (Table-2). The normalized gene expression was shown by the fold-change in the test sample. On the contrary, fold values >1 indicated a biologically significant approach (Figures-2 and 3). A *t*-test was performed to determine the significance of differences in the means for each gene between the treatment and control groups (Figures-1–3).

**Table-1:** List of genes and Plate-position for heart miRNAs in serum.

Mature ID	Plate-position	Fold change	95% CI	Rank
cfa-miR-361	3-F08	9.1899*	(2.34, 16.04)	260
cfa-miR-30e	3-B10	8.1107*	(2.08, 14.15)	214
cfa-miR-195	1-D04	0.0006	(0.00001, 0.00)	40
cfa-miR-369	4-A11	31.8023*	(0.00001, 87.79)	299
cfa-miR-652	3-D04	0.0069	(0.00001, 0.03)	232
cfa-miR-219-5p	3-B11	0.1866	(0.00001, 0.49)	215
cfa-miR-19b	3-D05	0.0505	(0.00001, 0.19)	233
cfa-miR-138a	2-E01	21.1401*	(0.00001, 47.80)	145
cfa-miR-133c	4-C01	0.0971	(0.00001, 0.30)	313
cfa-miR-142	4-A03	0.1527	(0.00001, 0.42)	291
cfa-miR-1	1-A06	4.4908*	(0.36, 8.62)	6
cfa-miR-502	3-F10	5.7905*	(0.00001, 13.92)	262
cfa-miR-425	3-B08	0.1579	(0.00001, 0.38)	212
cfa-miR-133a	1-B06	0.0313	(0.00001, 0.10)	18
cfa-miR-148b	3-C07	0.1166	(0.00001, 0.46)	223
cfa-miR-451	1-G06	0.2084	(0.00001, 0.44)	78
cel-miR-39-3p	3-H02	0.0148	(0.00001, 0.09)	278
cfa-miR-15a	1-C04	0.1848	(0.00001, 0.39)	28
cfa-miR-299	3-A04	0.066	(0.00001, 0.17)	100
cfa-miR-1271	4-B07	0.1868	(0.00001, 0.44)	307

* Upregulation. miRNAs=MicroRNAs, CI=Confidence interval

**Table-2:** Fold regulation for heart MiRNAs groups.

Plate-position	Mature ID	p-value (comparing to control group)	Fold regulation
3-F08	cfa-miR-361	0.000071	9.1899
3-B10	cfa-miR-30e	0.006345	8.1107
1-D04	cfa-miR-195	0.006487	–1765.0296
4-A11	cfa-miR-369	0.017785	31.8023
3-D04	cfa-miR-652	0.018131	–145.6454
3-B11	cfa-miR-219-5p	0.019579	–5.3578
3-D05	cfa-miR-19b	0.019694	–19.815
2-E01	cfa-miR-138a	0.020544	21.1401
4-C01	cfa-miR-133c	0.020914	–10.2973
4-A03	cfa-miR-142	0.021238	–6.5472
1-A06	cfa-miR-1	0.023642	4.4908
3-F10	cfa-miR-502	0.033211	5.7905
3-B08	cfa-miR-425	0.035337	–6.332
1-B06	cfa-miR-133a	0.038363	–31.9234
3-C07	cfa-miR-148b	0.039325	–8.5759
1-G06	cfa-miR-451	0.040414	–4.7986
3-H02	cel-miR-39-3p	0.042451	–67.7347
1-C04	cfa-miR-15a	0.043411	–5.4117
3-A04	cfa-miR-299	0.046191	–15.1577
4-B07	cfa-miR-1271	0.0478	–5.352

miRNAs=MicroRNAs

## Discussion

This study aimed to screen, profile, and characterize many induced miRNAs as biomarkers in dog hearts before and after experimentally inducing regional MIs by occluding the coronary arteries under general anesthesia and fluoroscopic guidance. The study results indicated that miRNA profiling was a good indicator of MI. At 2 h after MI, 20 biomarkers were found among 325 transcribed genes, 14 of which showed negative changes, suggesting downregulation, and six of which showed positive changes, indicating upregulation. Some of the previously described miRNAs in the heart and some novel miRNAs were found in these transcripts.

Endothelial dysfunction of the arterial wall is caused by inflammation of the arterial wall, hemodynamic abnormalities, and hypercholesterolemia. Etiological factors include infectious pathogens, homocysteine, and cigarette toxins. Atheroma develops due to endothelial dysfunction, generating remodeling, and inflammation in the artery wall, which leads to atherosclerosis [19]. The previous research has shown that severe shear stress considerably affects the gene expression in endothelial cells, and miRNAs play a role in atherosclerosis flow control [20]. The detected miRNA-361 is also a biomarker of heart failure in humans, as shown by its lower levels in heart failure, but it shows increased gene expression in AMI. In addition, miR-502 has been identified as a biomarker for CVD detection. The miRNA gene expression levels increased and positively correlated with NT-proBNP, indicating that increased serum concentrations reflect more severe cardiac malfunction [21]. The miR-93-5p biomarker has been identified as a gene that is a potential predictor of chronic pulmonary thromboembolic hypertension severity in humans and works by downregulating Alb and TP levels and upregulating uric acid, lactate dehydrogenase, and hydroxybutyrate dehydrogenase levels (p < 0.05). The miR-665 and miR-3202 genes have been previously reported to be upregulated and positively correlated with the NT-proBNP of miR-93-5p [22]. MiR-369, miR-219-5p, miR-19b, miR-133a (in MI), miR-652 (acute coronary syndromes), miR-142 (dual antiplatelet treatment), miR-425 (heart failure), and miR-451 (heart failure) are other well-known biomarkers of CVD [23–26]. On the other hand, MiR-148b reduces myocardial ischemia by modulating the Wnt/catenin signaling pathway, which leads to stem-cell regeneration [26].

Our study findings might be used in human medicine to assist in diagnosis, therapy, and evaluation of the treatment process, with the potential to reduce morbidity and death in patients. A potential study limitation is that because this was a preclinical animal investigation, the potential toxicity in humans might not be accurately predicted from this animal model. Another limitation was the small sample size. We also did not analyze the role of transcription factors in expression regulation.

## Conclusion

Profiling of miRNA before and after MI in a dog model showed upregulation of six novel biomarkers (3-F08, 3-B10, 4-A11, 1-A06, 2-E01, and 3-F10), which indicated various miRNA regulatory patterns. Gene expression was increased in the hearts of the dogs in the intervention group. These findings might be used as a basis for future human studies. These biomarkers can be clinically useful for individuals with AMI. These findings might also lead to more research on the potential use of miRNAs as biomarkers for MI.

## Authors’ Contributions

LAR: Conception, analysis, administration, and methods. KZA: Correspondence, analysis, and drafted the manuscript. MAMA, SAJ, and HH: Analysis and drafted the manuscript. All authors have read, reviewed, and approved the final manuscript.
